# A vegetable oil–based biopesticide with ovicidal activity against the two‐spotted spider mite, *Tetranychus urticae* Koch

**DOI:** 10.1002/elsc.202000042

**Published:** 2020-10-07

**Authors:** Naoki Takeda, Ayumi Takata, Yuka Arai, Kazuhiro Sasaya, Shimpei Noyama, Shigekazu Wakisaka, Noureldin Abuelfadl Ghazy, Dagmar Voigt, Takeshi Suzuki

**Affiliations:** ^1^ Graduate School of Bio‐Applications and Systems Engineering Tokyo University of Agriculture and Technology Koganei Tokyo Japan; ^2^ Kanagawa Agricultural Technology Center Hiratsuka Kanagawa Japan; ^3^ OAT Agrio Co., Ltd. Naruto Tokushima Japan; ^4^ Agriculture Zoology Department Faculty of Agriculture Mansoura University El‐Mansoura Egypt; ^5^ Japan Society for the Promotion of Science Chiyoda Tokyo Japan; ^6^ Institute for Botany Faculty of Biology Technische Universität Dresden Dresden Germany; ^7^ Institute of Global Innovation Research Tokyo University of Agriculture and Technology Koganei Tokyo Japan

**Keywords:** integrated pest management, natural enemies, *Neoseiulus californicus*, oil droplets, oil‐in‐water emulsion

## Abstract

A recently developed biopesticide made of safflower and cottonseed oils has excellent ovicidal activity against the hard‐to‐control spider mite *Tetranychus urticae* Koch (Acari: Tetranychidae). It has attracted attention as a sustainable treatment for controlling *T. urticae* because it has low potential for promoting resistance and little effect on the predatory mite *Neoseiulus californicus* (McGregor) (Acari: Phytoseiidae), which is an important natural enemy of spider mites. Here, we investigated the mechanism of its ovicidal activity against *T. urticae*. The oil droplets in the oil‐in‐water emulsion of the biopesticide strongly adhered to *T. urticae* eggs, seeped through the chorion being cut during hatching, and inhibited the embryonic rotational movement necessary for cutting and hatching. No adverse effect was observed on *N. californicus* eggs even in undiluted biopesticide. We conclude that this biopesticide and *N. californicus* can be used simultaneously in the integrated management of *T. urticae* in oily biopesticide‐tolerant plant species.

**Abbreviations**
CI: confidence interval
cryo‐SEM: cryo‐scanning electron microscopy
LC_50_: 50% lethal concentration
O/W: oil‐in‐water
PDSO: petroleum‐derived spray oil
SEM: scanning electron microscopy

## 1 INTRODUCTION

**FIGURE 1:**
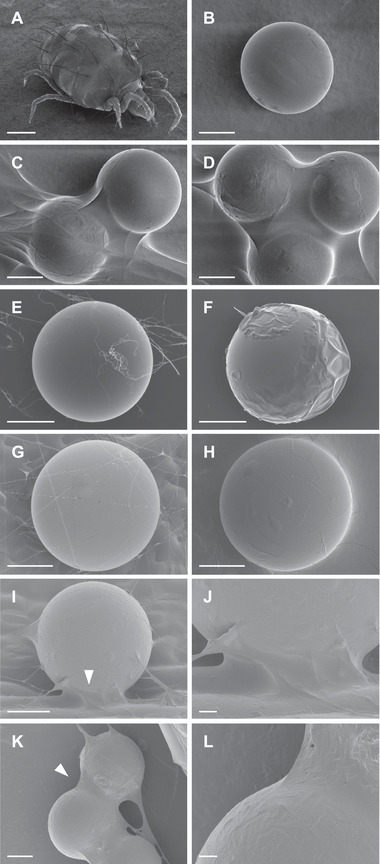
(A–F) SEM and (G–L) cryo‐SEM images of (A) adult female and (B–L) eggs and silk threads of *Tetranychus urticae*. Eggs were first dipped in (B, E, G) water (control) or (C, D, F, H–L) Suffoil^®^ diluted with water at 1:300 for 1 min. The oils establish liquid bridges between silk threads, eggs, and the substrate. (J, L) Details of areas indicated by white arrowheads in images to the left. Scale bars: (A) 100 µm, (B–I, K) 50 µm, (J, L) 10 µm

The two‐spotted spider mite, *Tetranychus urticae* Koch (Acari: Tetranychidae) (Figure 1A), is a cosmopolitan agricultural pest that feeds on more than 1100 plant species [1]. Synthetic pesticides have long been used to control it. However, because of its short developmental time and high reproductive potential, *T. urticae* has developed resistance to 96 active ingredients [2, 3]. Among growing public concern about the environmental and health risks associated with the excessive use of synthetic pesticides, the development of alternative treatments which are less prone to the development of resistance and are less harmful to human and environmental health, is urgently required.

Food ingredients and food additives (e.g., hydroxypropyl starch, propylene glycol esters of fatty acids) have been used as alternative pesticides that physically inhibit respiration and behaviors of pest arthropod herbivores [4, 5]. The major advantage of these products over the chemically acting conventional synthetic pesticides is the elimination of concerns over the development of resistance because of their physical pesticidal properties. Although the same advantage can be found in petroleum‐derived spray oils (PDSOs) [6], food ingredients and food additives are less toxic to mammals and can be used for pest control without compromising environmental health.

Suffoil^®^ is a biopesticide made from 97% v/v safflower and cottonseed oils and 3% v/v emulsifier, and forms oil‐in‐water O/W emulsions. It has acaricidal activity against all developmental stages of *T. urticae*. Unlike most pesticides that contain food ingredients or food additives, Suffoil^®^ has excellent ovicidal (egg‐killing) activity against *T. urticae* [7], and thus offers effective control. In addition, it has little effect on the predatory mite *Neoseiulus californicus* (McGregor) (Acari: Phytoseiidae), which is an important natural enemy of spider mites. *Neoseiulus californicus* is sold commercially to manage populations of *T. urticae* and other spider mites in greenhouses and open fields [8, 9]. Therefore, the implementation of both the biopesticide and predatory mites in integrated pest management programs is highly anticipated. However, the mode of action of the biopesticide and the reason why it has different activities against eggs of *T. urticae* and *N. californicus* remain unclear.

Here, we investigated the mechanism of the ovicidal activity of the biopesticide against *T. urticae* and *N. californicus* eggs by microscopy and experiments.

## 2 MATERIALS AND METHODS

### 2.1 Mites

Two populations of *T. urticae* were used. One population was established in London, Ontario, Canada, in the early 2000s and was used for whole‐genome sequencing [10]. The other was established in Dresden, Saxony, Germany, in 2012 from individuals collected from strawberry crops in Weixdorf, near Dresden, and was used for the study of plant–spider mite interactions [11, 12]. The London population was maintained on seedlings of kidney bean (*Phaseolus vulgaris* L. ‘Hatsumidori #2′, Takii & Co., Ltd., Kyoto, Japan) at an air temperature of 24 to 26°C, a relative humidity (RH) of 50% to 65%, and a light period of 16 h day^−1^. The Weixdorf population was maintained on seedlings of broad bean (*Vicia faba* L. ‘Witkiem’; ISP‐International Seeds Processing GmbH, Quedlinburg, Germany) at 22 to 24°C, 50% to 65% RH, and a light period of 16 h day^−1^ in wooden, gauze‐covered rearing cages. A commercial population of *N. californicus* was obtained from Sumika Technoservice Corp. (Takarazuka, Japan) in 2017 and maintained under laboratory conditions on detached kidney bean leaves infested with *T. urticae* in ventilated plastic boxes.

**PRACTICAL APPLICATION**

The high ovicidal activity of a vegetable oil–based biopesticide against spider mites is due to the inhibition of hatching behavior. Interestingly, no ovicidal effect was observed on the phytoseiid mites used as biological control agents against spider mites. This finding will greatly contribute to the use of the biopesticide in integrated pest management programs. The method used to evaluate ovicidal activity in this study can be used to screen other pesticides that target the hatching behavior of spider mites.

### 2.2 Biopesticide preparation

Suffoil^®^ (OAT Agrio Co., Ltd., Tokyo, Japan) was mixed with water at a ratio of 1:30, 1:300 (manufacturer's recommended ratio), 1:3000, or 1:30 000 and emulsified in a Vortex‐Genie 2 mixer (Scientific Industries, Inc., Bohemia, NY, USA). Water and paraffin oil (#164‐00476; Fujifilm Wako Pure Chemical Corp., Osaka, Japan), both harmless to spider mite eggs [13, 14], were used as controls.

### 2.3 Scanning electron microscopy (SEM) and cryo‐SEM

For high‐resolution observations of treated *T. urticae* eggs, we used SEM with a VHX‐D500 microscope (Keyence Corp., Osaka, Japan) and a JSM‐6510 microscope (JEOL Ltd., Tokyo, Japan), and cryo‐SEM with an FE‐SEM Supra 40VP‐31‐79 microscope (Carl Zeiss SMT, Oberkochen, Germany) equipped with an Emitech K250X cryo‐preparation unit (Quorum Technologies Ltd., Ashford, Kent, UK).

Adult *T. urticae* females were placed onto fresh *P. vulgaris* ‘Hatsumidori #2′ leaves with a fine tapered artist's brush and allowed to lay eggs for 24 h. The eggs laid on the leaves were gently detached and transferred to one side of a double‐sided adhesive carbon tape (Nisshin EM Co.,Ltd., Tokyo, Japan). The tape with eggs was immersed in water (control) or 1:300 Suffoil^®^ for 1 min and then air‐dried for up to 3 h. The side of the tape opposite the eggs was attached to a metal stub. The samples with no sputtering treatment were examined by SEM with the VHX‐D500 microscope at 1.2 kV. Other treated samples were coated with a 20‐nm layer of gold in an ion sputter coater (IB‐3; Eiko Engineering, Ltd., Tokyo, Japan) and examined by higher resolving SEM with the JSM‐6510 microscope at 10 kV.

*Tetranychus urticae*–infested *V. faba* ‘Witkiem’ leaves were immersed in water (control) or 1:300 Suffoil^®^ for 1 min and air‐dried for up to 3 h. The treated leaves were cut into 5‐mm × 5‐mm pieces with micro‐scissors and mounted on metal holders in polyvinyl alcohol Tissue‐Tek O.C.T. compound (Sakura Finetek Europe BV, Alphen aan den Rijn, The Netherlands). They were shock‐frozen in liquid nitrogen, transferred to the cryo‐preparation chamber at −140°C, sublimed for 30 min at −70°C, sputter‐coated with platinum (∼6 nm), transferred to the cryo‐SEM, and then examined at −100°C at 5 kV.

### 2.4 Biopesticide dose‐response of egg hatching

Adult *T. urticae* females were placed in an arena fenced with wet Kimwipes (S‐200; Nippon Paper Crecia Co., Ltd., Tokyo, Japan) in a 35‐mm‐diameter polystyrene Petri dish (75 mites dish^−1^) and were allowed to lay eggs for 24 h. All females and wet Kimwipes were then removed, and the deposited eggs were incubated at 25°C and 100% RH for 4 days to synchronize embryonic development just before hatching [13]. Adult *N. californicus* females were placed in an arena fenced with Tangle B adhesive (Fuji Yakuhin Kogyo K.K., Tokyo, Japan) in a 35‐mm‐diameter Petri dish (40 to 150 mites dish^−1^) and allowed to lay eggs for 24 h. Synchronized 5‐day‐old *T. urticae* eggs and 1‐day‐old *N. californicus* eggs were used in the following experiments.

Water (control) or Suffoil^®^ diluted with water at 1:300, 1:3000, or 1:30 000 was added into the Petri dishes in which the *T. urticae* or *N. californicus* eggs were prepared. The eggs were immersed for 1 min and then drained. Eggs were incubated at 25°C and 50% RH and egg hatch were assessed after 24 and 48 h with a SZ51 stereomicroscope (Olympus Corp., Tokyo, Japan). The assay was conducted in three independent experimental runs.

### 2.5 Biopesticide effect on embryonic development

In a preliminary test to confirm the time to eye formation in *T. urticae* embryos, a single egg immersed in paraffin oil was photographed with a DFC500 digital camera installed on a DM2500 light microscope (both from Leica Microsystems GmbH, Wetzlar, Germany) every 30 min.

Adult *T. urticae* females were placed in an arena fenced with wet Kimwipes in a 35‐mm‐diameter Petri dish (25 mites dish^−1^) and allowed to lay eggs for 24 h. After 24 h, all females and wet Kimwipes were removed from the dish, and 2‐mL of water (control) or 1:300 Suffoil^®^ was added. The eggs were immersed for 1 min and then drained. Eggs were incubated at 25°C and 50% RH and embryonic formation of eyes and the hatching of larvae from treated eggs were observed and recorded every day through the stereomicroscope. The assay was conducted in three independent experimental runs.

### 2.6 Quantification of biopesticide coverage of mite eggs

Oil Red O (#154‐02072; Fujifilm Wako Pure Chemical Corp.) used as a tracer dye was added to Suffoil^®^ at 200 mM w/v to give a final dye concentration of 6.7 ppm (1:30 dilution of Suffoil^®^), 6.7 × 10^−1^ (1:300), or 6.7 × 10^−2^ ppm (1:3000). One‐day‐old *T. urticae* and *N. californicus* eggs were immersed in 2‐mL of biopesticide in a 35‐mm‐diameter Petri dish for 1 min. The drained eggs were then photographed with a digital camera (EOS Kiss X7; Canon Inc., Tokyo, Japan) installed on a high‐magnification stereomicroscope (M205FA; Leica Microsystems GmbH). The proportion of red in the pixels of an egg in each image was evaluated in ImageJ 2 software [15] to quantify the biopesticide coverage on the surface of the egg.

### 2.7 Effects of biopesticide immersion and rinsing on egg hatching

Synchronized 5‐day‐old *T. urticae* eggs (see section 2.4) and 1‐day‐old *N. californicus* eggs were immersed in 2 mL paraffin oil (control) or undiluted Suffoil^®^ in a 35‐mm‐diameter Petri dish and incubated at 25°C. Egg hatch was observed with the stereomicroscope at 24 and 48 h after treatment. The assay was conducted in three independent experimental runs.

Synchronized 5‐day‐old *T. urticae* eggs were immersed in 2 mL of dyed 1:300 Suffoil^®^ (see section 2.6) in a 35‐mm‐diameter Petri dish at 25°C for 1 min. The liquid was then drained from the dish; some of the treated eggs were then rinsed with paraffin oil. The eggs were incubated at 25°C for 24 h, and then egg hatch was observed. The assay was conducted in three independent experimental runs.

### 2.8 Data analysis

All data were analyzed in *R* v. 3.3.2 software [16]. Dose–response relationships in the pesticide assay were detected and curves were generated with the two‐parameter log‐logistic function (*drm*() function in the *R* package ‘*drc*’); they are presented with a 95% confidence interval (CI). Time‐course curves of the proportion of eggs containing an embryo with visible eye spots and hatched larvae of *T. urticae* were plotted with the Kaplan–Meier method (*survfit*() function in the *R* package ‘*survival*’). Differences in curves between control and biopesticide treatments were analyzed by the log‐rank test (*survdiff*() function in the *R* package ‘*survival*’). Proportional data on biopesticide coverage of mite eggs and the hatching of larvae from eggs immersed in undiluted biopesticide and rinsed with paraffin oil were arcsine‐square‐root transformed, and the normalized data were analyzed by ANOVA (*aov*() function in the *R* package ‘*stats*’) followed by Tukey's HSD test (*glht*() function in the *R* package ‘*multcomp*’) or *t*‐test (*t.test*() function in the *R* package ‘*stats*’) of differences between species and treatments. Results of the oil‐covered area on eggs and the hatch rate of eggs treated with paraffin oil and biopesticide, or after rinsing with paraffin oil, are presented as overlaid bee‐swarm (*beeswarm*() function in the *R* package ‘*beeswarm*’) and box‐and‐whisker plots (*boxplot*() function in the *R* package ‘*graphics*’).

## 3 RESULTS

### 3.1 The spider mite egg–oily substance interface

SEM and cryo‐SEM images clearly demonstrated the biopesticide covering the surface of eggs and the surrounding silk threads, creating liquid bridges between threads, eggs, and substrate (Figure 1). Some eggs were completely covered; others showed patches (Figure 1F). The silk threads appeared to act as a scaffold for biopesticide contact. Obviously, their polymeric structure altered/denaturated after oil wetting. Also, the biopesticide accumulated between the egg and the leaf (Figure 1I, J).

### 3.2 Hatching behavior (without treatment)

*Tetranychus urticae* larvae typically rotated inside the spherical egg and probably cut the chorion with their appendages during the rotational movement before they hatched (Supplementary Movies S1, S3). *Neoseiulus californicus* larvae did not; instead, larvae in the ellipsoid egg first penetrated the chorion with their dorsal setae Z5 and then extend their legs for hatching (Supplementary Movie S2).

### 3.3 Biopesticide dose–response of egg hatching

**FIGURE 2:**
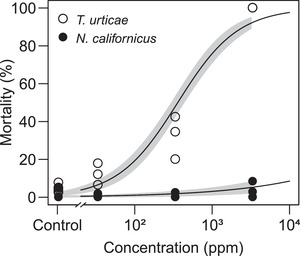
Dose–response of hatching in *Tetranychus urticae* and *Neoseiulus californicus*. Eggs were dipped in water (control) or Suffoil^®^ diluted with water at 1:30 000 (3.2 × 10^1^ ppm), 1:3000 (3.2 × 10^2^ ppm), or 1:300 (3.2 × 10^3^ ppm) for 1 min. Drained eggs were incubated at 25°C. Data were collected from three independent experimental runs and fitted to a regression curve with a 95% CI (shown by gray area). Numbers of eggs used in each independent experimental run were 60 to 70 (control), 31 to 101 (3.2 × 10^1^ ppm), 58 to 80 (3.2 × 10^2^ ppm) and 45 to 86 (3.2 × 10^3^ ppm) for *T. urticae*, and 30 to 40 (control), 35 to 66 (3.2 × 10^1^ ppm), 20 to 41 (3.2 × 10^2^ ppm) and 25 to 36 (3.2 × 10^3^ ppm) for *N. californicus*. The LC_50_ value for *T. urticae* eggs was 359 ± 37 ppm (mean ± SE)

The biopesticide caused a dose‐dependent increase in lethality to *T. urticae* eggs (Figure 2). The LC_50_ value was 359 ± 37 ppm (mean ± SE, *n* = 31 to 101). In contrast, it had almost no lethality to *N. californicus* eggs: the mortality was < 10% (*n* = 20 to 66), even at the manufacturer's recommended dilution ratio of 1:300.

### 3.4 Biopesticide effect on embryonic development

**FIGURE 3:**
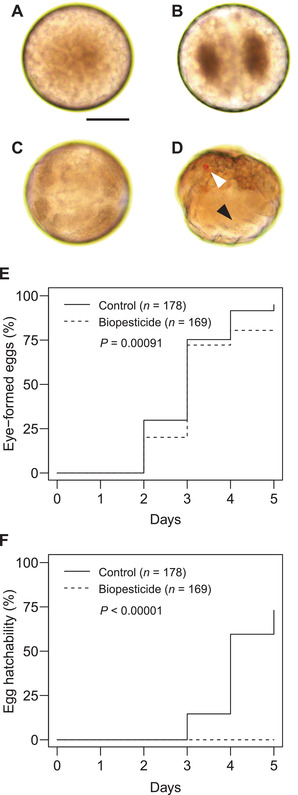
Embryonic development in *Tetranychus urticae* eggs immersed in paraffin oil (A) 2, (B) 3, (C) 25, and (D) 53 h after oviposition and held at 25°C. (A) Uncleaved egg filled with yolk. (B) Two‐cell stage established by first cleavage division. (C) Early stage of embryonic appendage formation. (D) Eye‐spot stage. White arrowhead, eyes; black arrowhead, appendages. Scale bar: 50 µm. (E, F) Time series of (E) proportion of eggs at the eye‐spot stage or later and (F) hatchability of *T. urticae* larvae after being dipped in water (control) or Suffoil^®^ diluted with water at 1:300 for 1 min at 25°C. Drained eggs were incubated at 25°C. Data were collected from three independent experimental runs. Time‐course curves were plotted according to the Kaplan–Meier method and compared by log‐rank test

Eyes formed (Figure 3D) in > 80% of eggs (*n* = 169) even at 1:300 Suffoil^®^ (Figure 3E), only slightly less than in the control (>90%, *n* = 178). Despite this, the hatchability was 0% (*n* = 169), and there was a significant difference in the time‐course curves between the biopesticide treatment and the control (Figure 3F).

### 3.5 Biopesticide coverage on mite eggs

**FIGURE 4:**
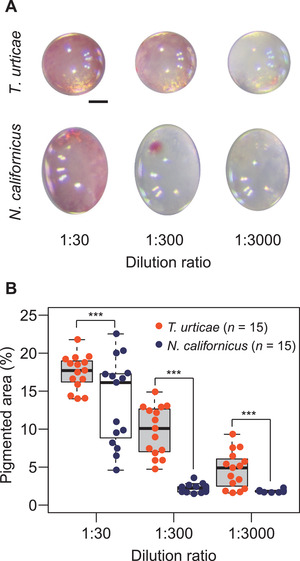
Visualization and quantification of oils on mite eggs by use of a lipophilic red dye. (A) *Tetranychus urticae* and *Neoseiulus californicus* eggs were dipped for 1 min in Suffoil^®^ containing 200 mM (w/v) Oil Red O, diluted 1:30, 1:300, or 1:3000 with water. Images of drained eggs were taken with a digital camera attached to a stereomicroscope. (B) The proportion of red in the pixels within a single egg in each RGB image was evaluated as the amount of biopesticide on eggs. Individual data are displayed as a bee‐swarm. In the box‐and‐whisker plots, the central line indicates the median, the distance between the box bottom (first quartile) and top (third quartile) corresponds to the interquartile range, and the whiskers indicate the minimum and maximum values. ***p < 0.001, unpaired *t*‐test of normalized data

A significantly larger area of each egg chorion surface was covered by the biopesticide in *T. urticae* (*n* = 15) than in *N. californicus* (*n* = 15) at 1:30, 1:300, and 1:3000 dilution ratios (Figure 4).

### 3.6 Effects of biopesticide immersion and rinsing on egg hatching

**FIGURE 5:**
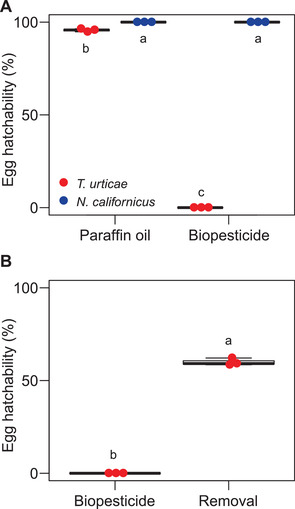
Effects of oil immersion and rinsing on egg hatch. (A) Hatching from *Tetranychus urticae* and *Neoseiulus californicus* eggs immersed in paraffin oil (control) or undiluted Suffoil^®^ (biopesticide) in a Petri dish at 25°C. Data were collected from three independent experimental runs. Numbers of eggs used in each independent experimental run were 56 to 134 (paraffin oil) and 53 to 110 (biopesticide) for *T. urticae*, and 20 to 26 (paraffin oil) and 19 to 23 (biopesticide) for *N. californicus*. (B) Hatching from *T. urticae* eggs treated with dyed Suffoil^®^ (1:300) before (control) and after rinsing with paraffin oil. Data were collected from three independent experimental runs. Numbers of eggs used in each independent experimental run were 37 to 71. The different letter indicates a significant difference at *p *< 0.05 by (A) ANOVA followed by Tukey's HSD test and (B) paired *t*‐test of normalized data. Figure 4 for explanations of the bee‐swarm and box‐and‐whisker plots

Almost all eggs of *T. urticae* (*n* = 56 to 134) and *N. californicus* (*n* = 20 to 26) hatched when immersed in paraffin oil, although significantly lower hatchability in *T. urticae* than in *N. californicus* (Figure 5A). When immersed in the undiluted Suffoil^®^, although no larvae of *T. urticae* hatched (*n* = 53 to 110), almost 100% of *N. californicus* larvae hatched (*n* = 19 to 23). Rinsing with paraffin oil to remove the dyed 1:300 Suffoil^®^ significantly increased the hatchability in *T. urticae* to about 60% (*n* = 37 to 71) (Figure 5B).

## 4 DISCUSSION

High‐resolution observations by SEM and cryo‐SEM showed that the Suffoil^®^ adhered to the surface of eggs and to the surrounding web, forming a coat‐like layer (Figure 1C, D, F, I–L). Walking larvae to adults of tetranychid mites continuously produce webs by excreting silk through orifices in their pedipalps which are the second pair of appendages [17]. The amino acid composition of the silk is rich in glycine, glutamic acid, alanine, serine, and aspartic acid, which account for ∼60% of the total, and resembles mammalian pre‐keratin, a fibrous protein [18]. The silk fibers of *T. urticae* are transparent and have a diameter of 54 nm (adult silk) and 23 nm (larval silk) [10], and are thus thinner than the silk fibers of spiders [19] and silkworms [20]. The eggs of the carmine spider mite, *Tetranychus cinnabarinus* (Boisduval), a red form of *T. urticae* [21], are laid on or under the webbing [17], which may protect them against the environment and natural enemies. The strong adhesion of the biopesticide to the *T. urticae* silk threads (Figure 1C, D, F, I–L) suggests a high affinity of the oils or the surfactant for the bipolar silk proteins. This high affinity may support the high ovicidal activity of the biopesticide, because *T. urticae* eggs are also laid on or under webbing [17].

The manufacturer's recommended dilution ratio of Suffoil^®^ had high ovicidal activity against *T. urticae* but almost no effect on *N. californicus* eggs (Figure 2). The area of oily coverage was greater on *T. urticae* eggs than on *N. californicus* eggs (Figure 4), perhaps because *N. californicus*, unlike *T. urticae*, does not produce silk threads with a high affinity for the biopesticide and the material properties of chorion could differ between phytophagous and predatory mites. Although the difference in the amount of biopesticide adhering to eggs could be the reason for the difference in the ovicidal activity between species, almost 100% of *N. californicus* larvae hatched, even when the eggs were immersed in the undiluted biopesticide (Figure 5A). Since the biopesticide has no ovicidal activity against *N. californicus*, we conclude that both Suffoil^®^ and *N. californicus* can be applied simultaneously for the environmentally friendly and sustainable management of *T. urticae* in oil‐tolerant plant species.

Respiration is essential for *T. urticae* eggs; they die under anoxic (0% O_2_) conditions at 25°C for > 8 h [22, 23]. Since two perforation organs penetrate the *T. urticae* chorion for taking in air [24], it was initially considered that biopesticides would block these organs and suffocate the eggs. Suffocation by spiracle/stigma blockage was the most accepted theory of the mode of action of PDSOs [25]. To verify this hypothesis, we investigated embryogenesis in eggs after biopesticide treatment. Spider mites have a pair of eyes [26] that are formed late in embryogenesis and are visible through the transparent chorion because of the red pigmentation (Figure 3D), which is a good indicator of healthy embryonic development. As eyes were formed in > 90% of eggs in the control and in > 80% eggs treated with the biopesticide (Figure 3E), the biopesticide has a slight negative effect on embryogenesis, probably by respiratory inhibition. However, no larvae hatched from the biopesticide‐treated eggs, despite their readiness to hatch (Figure 3F).

**FIGURE 6:**
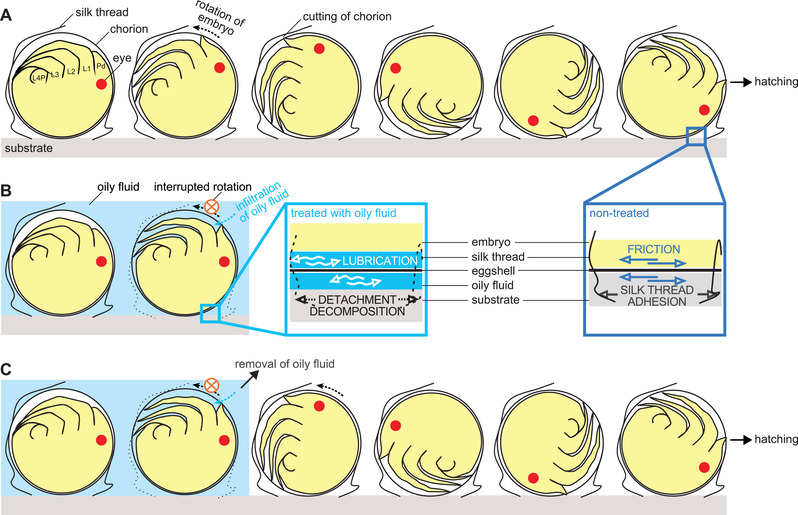
Suggested schematic process of hatching from egg in *Tetranychus urticae* (A) under untreated condition, (B) after treatment with Suffoil^®^, subsequent infiltration into the egg, and silk thread alteration, and (C) after treatment with Suffoil^®^ and subsequent removal of Suffoil^®^. The egg may loosely lie on the substrate, or be attached to silk threads which adhere to the substrate. The embryo at the pharate‐larva stage rotates to cut the egg chorion for hatching. Without any treatment (details in the dark blue square), we conjecture friction occurring between embryo and inner chorion during the rotational movement. The egg is, however, reliably attached to the substrate by frictional forces acting at the outer chorion‐substrate interface and/or the adhesive fixation by silk threads. After treatment with oily fluid (light blue background in B, C, and detail in the light blue square), the fluid not only seeps into the egg through the cut chorion but possibly decomposes silk threads (dotted lines) which loose their adhesive properties. Thus, friction‐based repulsion required for the rotational movement towards hatching is interfered probably by oil lubrication between the chorion and embryo and because the oily fluid leads to silk decomposition and the detachment of egg‐fixing silk threads from the substrate. Interestingly, the impact of oily fluid is recoverable when the oil is subsequently removed (C). Pd, pedipalps; L1–3, first, second, and third pair of walking legs; L4P, primordia of the fourth pair of walking legs [28]

Once embryogenesis is completed, the spider mite embryo hatches by cutting the chorion probably with the appendages while rotating within the egg (Supplementary Movies S1, S3; Figure 6A) [27]. However, in the biopesticide‐treated eggs, no rotational movement was observed, or the movement was interrupted (Supplementary Movies S4, S5, S6, Figure 6B). The red dye clearly identified oil droplets (∼10 to 50 µm diameter) in the O/W emulsion (1:300), which drifted as the moisture evaporated and adhered to the egg and surrounding silk threads of webbing (Supplementary Movie S7). Hatching increased after the removal of the biopesticide (Figure 5B, 6C), supporting the theory that the oily coverage inhibits the hatching behavior of *T. urticae* larvae.

Taken together, these results show that oil droplets in the O/W emulsion of the biopesticide effectively adhered to *T. urticae* eggs and silk threads, establishing liquid bridges between threads, eggs, and substrate. The oils seeped into the eggs through the cut chorion as the larvae were hatching and inhibited the embryonic rotational movement essential for hatching (Figure 6A). Therefore, it may act mainly by inhibiting the hatching mechanics. We suggest that it causes a loss of repulsion of moving larvae inside the egg by creating a lubrication layer (Figure 6B). In addition, molecular interaction with the oils could alter the anchoring silk threads, decreasing their adhesion on the substrate, and thus, suppressing the internal rotations of the larvae. Under untreated conditions, the eggs would be reliably attached to the substrate by silk threads, providing a stable capsule in which larvae may efficiently rotate because of repulsion generated by friction between the larval integument and the inner chorion.

On the other hand, no adverse effect of the biopesticide was observed on *N. californicus* eggs. This may be due to the fact that the hatching behavior of *N. californicus* is completely different from that of *T. urticae* and does not show any rotational movement inside the eggs (Supplementary Movie S2).

Further studies are required to fully clarify why *N. californicus* eggs are resistant to the biopesticide. Also, detailed understanding of molecular interactions occurring during diffusion of the biopesticide through the chorion is needed to evaluate the overall effects in *T. urticae* eggs under varying environmental conditions.

## CONFLICT OF INTEREST

This study was funded by OAT Agrio Co., Ltd., which employs Shimpei Noyama and Shigekazu Wakisaka.

## Supporting information


**Movie S1**. Hatching behavior of a *Tetranychus urticae* larva in untreated egg. The larva rotates in the spherical egg to cut the chorion for hatching; 32 × accelerated.
**Movie S2**. Hatching behavior of *Neoseiulus californicus* larvae in untreated eggs. The larvae in the ellipsoid eggs first penetrate the eggshell with their dorsal setae Z5 and then extend their legs for hatching; 16 × accelerated.
**Movie S3**. *Tetranychus urticae* eggs treated with water (control); 128 × accelerated.
**Movie S4**. *Tetranychus urticae* eggs treated with 1:300 Suffoil^®^; 128 × accelerated.
**Movie S5**. *Tetranychus urticae* egg treated with 1:300 Suffoil^®^ stained with Oil Red O. The accumulation of red dye in the egg over time indicates that the biopesticide seeped into the egg through the cut chorion; 128 × accelerated.
**Movie S6**. *Tetranychus urticae* egg treated with 1:300 Suffoil^®^ stained with Oil Red O. When the cutting of the chorion by the rotational movement of the embryo reaches the bottom of the egg, where the biopesticide accumulated (Figure 1I, J), the oil infiltrates the egg and the rotational movement stops; 128 × accelerated.
**Movie S7**. Suffoil^®^ stained with Oil Red O is clearly identified as oil droplets in the O/W emulsion, which drifted as the moisture evaporated and adhered to the egg and surrounding silk threads; 128 × accelerated.
**Movies are available at the following link**:
https://doi.org/10.6084/m9.figshare.12895292
