# Supplementary material for: A vegetable oil–based biopesticide with ovicidal activity against the two‐spotted spider mite, Tetranychus urticae Koch
Source: Eng Life Sci. 2020 Oct 7;20(11):525–34. doi: 10.1002/elsc.202000042 (PMC7645644; doi:10.1002/elsc.202000042)
Supplement: Supplementary file 1 — Movie S1. Hatching behavior of a Tetranychus urticae larva in untreated egg. The larva rotates in the spherical egg to cut the chorion for hatching; 32 × accelerated. Movie S2. Hatching behavior of Neoseiulus californicus larvae in untreated eggs. The larvae in the ellipsoid eggs first penetrate the eggshell with their dorsal setae Z5 and then extend their legs for hatching; 16 × accelerated. Movie S3. Tetranychus urticae eggs treated with water (control); 128 × accelerated. Movie S4. Tetranychus urticae eggs treated with 1:300 Suffoil®; 128 × accelerated. Movie S5. Tetranychus urticae egg treated with 1:300 Suffoil® stained with Oil Red O. The accumulation of red dye in the egg over time indicates that the biopesticide seeped into the egg through the cut chorion; 128 × accelerated. Movie S6. Tetranychus urticae egg treated with 1:300 Suffoil® stained with Oil Red O. When the cutting of the chorion by the rotational movement of the embryo reaches the bottom of the egg, where the biopesticide accumulated (Figure 1I, J), the oil infiltrates the egg and the rotational movement stops; 128 × accelerated. Movie S7. Suffoil® stained with Oil Red O is clearly identified as oil droplets in the O/W emulsion, which drifted as the moisture evaporated and adhered to the egg and surrounding silk threads; 128 × accelerated. Movies are available at the following link: https://doi.org/10.6084/m9.figshare.12895292 [file ELSC-20-525-s001.docx]

# Supporting information

**Movie S1.** Hatching behavior of a *Tetranychus urticae* larva in untreated egg. The larva rotates in the spherical egg to cut the chorion for hatching; 32× accelerated.

**Movie S2.** Hatching behavior of *Neoseiulus californicus* larvae in untreated eggs. The larvae in the ellipsoid eggs first penetrate the eggshell with their dorsal setae Z5 and then extend their legs for hatching; 16× accelerated.

**Movie S3.** *Tetranychus urticae* eggs treated with water (control); 128× accelerated.

**Movie S4.** *Tetranychus urticae* eggs treated with 1:300 Suffoil^®^; 128× accelerated.

**Movie S5.** *Tetranychus urticae* egg treated with 1:300 Suffoil^®^ stained with Oil Red O. The accumulation of red dye in the egg over time indicates that the biopesticide seeped into the egg through the cut chorion; 128× accelerated.

**Movie S6.** *Tetranychus urticae* egg treated with 1:300 Suffoil^®^ stained with Oil Red O. When the cutting of the chorion by the rotational movement of the embryo reaches the bottom of the egg, where the biopesticide accumulated (Figure 1I, J), the oil infiltrates the egg and the rotational movement stops; 128× accelerated.

**Movie S7.** Suffoil^®^ stained with Oil Red O is clearly identified as oil droplets in the O/W emulsion, which drifted as the moisture evaporated and adhered to the egg and surrounding silk threads; 128× accelerated.

**Movies are available at the following link:**

https://doi.org/10.6084/m9.figshare.12895292
